# Use of a handheld Doppler to measure brachial and femoral artery occlusion pressure

**DOI:** 10.3389/fphys.2023.1239582

**Published:** 2023-08-17

**Authors:** Pat R. Vehrs, Shay Richards, Chase Blazzard, Hannah Hart, Nicole Kasper, Ryan Lacey, Daniela Lopez, Luke Baker

**Affiliations:** ^1^ Department of Exercise Sciences, Brigham Young University, Provo, UT, United States; ^2^ Department of Statistics, Ohio State University, Columbus, OH, United States

**Keywords:** arterial occlusion pressure, Doppler ultrasound, occlusion training, KAATSU, blood flow restriction

## Abstract

**Objective:** Measurement of arterial occlusion pressure (AOP) is essential to the safe and effective use of blood flow restriction during exercise. Use of a Doppler ultrasound (US) is the “gold standard” method to measure AOP. Validation of a handheld Doppler (HHDOP) device to measure AOP could make the measurement of AOP more accessible to practitioners in the field. The purpose of this study was to determine the accuracy of AOP measurements of the brachial and femoral arteries using an HHDOP.

**Methods:** We simultaneously measured AOP using a “gold standard” US and a HHDOP in the dominant and non-dominant arms (15 males; 15 females) and legs (15 males; 15 females).

**Results:** There were no differences in limb circumference or limb volume in the dominant and non-dominant arms and legs between males and females or between the dominant and non-dominant arms and legs of males and females. The differences between US and HHDOP measures of AOP in the dominant and non-dominant arms and legs were either not significant or small (<10 mmHg) and of little practical importance. There were no sex differences in AOP measurements of the femoral artery (*p* > 0.60). Bland–Altman analysis yielded an average bias (−0.65 mmHg; −2.93 mmHg) and reasonable limits of agreement (±5.56 mmHg; ±5.58 mmHg) between US and HHDOP measures of brachial and femoral artery AOP, respectively.

**Conclusion:** HHDOP yielded acceptable measures of AOP of the brachial and femoral arteries and can be used to measure AOP by practitioners for the safe and effective use of blood flow restriction. Due to the potential differences in AOP between dominant and non-dominant limbs, AOP should be measured in each limb.

## Introduction

Use of blood flow restriction (BFR) during exercise provides positive outcomes for load-compromised individuals. The appropriate use of a pneumatic cuff during BFR partially restricts arterial blood flow into the muscle and occludes venous blood flow out of the muscle (Pope et al., 2013; Iida et al., 2005; Iida et al., 2007; Loenneke and Pujol, 2009; Scott et al., 2015). Current recommendations for the safe and effective use of BFR include using an individualized cuff pressure of 40%–80% of the limb’s arterial occlusion pressure (AOP) to restrict arterial blood flow (Scott et al., 2015; Hughes et al., 2017; McEwen et al., 2019; Patterson et al., 2019; Cognetti et al., 2022). Use of occlusion pressures at the lower end of this range (i.e., 40%–60% of the limb’s AOP) may help avoid some of the deleterious effects of regular use of higher pressures (Nascimento et al., 2019). Previous research indicates that the limb’s AOP is related to the circumference of the limb (Scott et al., 2015; McEwen et al., 2019; Hughes et al., 2017; Jessee et al., 2016a; Tafuna’i et al., 2021; Mattocks et al., 2018) and other factors, such as systolic blood pressure (Loenneke et al., 2015; Brown et al., 2018), mean arterial pressure (Vehrs et al., 2023), width of the occlusion cuff (Loenneke et al., 2012), placement of the cuff on the limb (Spitz et al., 2020), limb dominance (Tafuna’i et al., 2021), body position (Karanasios et al., 2021), and gender (Tafuna’i et al., 2021; Brown et al., 2018; Vehrs et al., 2023). Because various factors affect the limb’s AOP, an individualized approach to the use of BFR during exercise is recommended (Scott et al., 2015; McEwen et al., 2019; Patterson et al., 2019; Spitz et al., 2022). Use of non-personalized methods of setting BFR pressures are not recommended due to the application of unknown pressures, lack of control of blood flow restriction, safety issues, and the inconsistent stimuli between exercise sessions (McEwen et al., 2019). The risks of adverse outcomes (e.g., numbness, dizziness, subcutaneous hemorrhage, nerve injury, rhabdomyolysis, and thromboembolism) associated with BFR increase with the use of high cuff pressures that occlude rather than restrict arterial blood flow (Anderson et al., 2022). Thus, the measurement of the limb’s AOP is essential to the safe and effective use of BFR during exercise.

Use of a Doppler ultrasound (US) is the “gold standard” method to measure AOP because the direction of blood flow can be detected and the velocity and volume (mL/min) of blood flow can be measured. The US can also display a pulse wave. Using the US, AOP is defined as the lowest cuff pressure at which the pulse wave and arterial blood flow are no longer detectable (Tafuna’i et al., 2021; Vehrs et al., 2023; Crossley et al., 2019). Doppler ultrasound machines are used in some clinical and research settings, but their high cost and required training often prohibit their use by athletic trainers, strength and conditioning coaches, personal trainers, and many clinicians (e.g., physical therapists). Use of relatively inexpensive hand-held devices can make the safe and effective use of BFR during exercise more accessible to many professionals. A handheld Doppler (HHDOP) can detect blood flow and give an audible signal corresponding to each pulse wave. Previously reported data comparing measures of AOP using a US and an HHDOP are promising. For example, HHDOP (133 ± 18 mmHg) and US (135 ± 117 mmHg) measures of femoral artery AOP in young college-aged males were similar (Laurentino et al., 2020). Nevertheless, these data should be interpreted with caution since close examination of data (Laurentino et al., 2020) reveals Bland–Altman plots with wide limits of agreement. One explanation for some of the bias between the two measures of AOP is that the two measures were taken at different times. Additional research comparing measures of AOP using a “field” HHDOP device to those of a “gold standard” US device is warranted. In addition, we are not aware of studies that have compared simultaneous US and HHDOP measures of AOP of the brachial and femoral arteries of males and females.

Although numerous previous studies have included male and female participants (Mouser et al., 2017a; Ingram et al., 2017; Mattocks et al., 2017; Hughes et al., 2018a; Bell et al., 2018; Mouser et al., 2018; Sieljacks et al., 2018; Crossley et al., 2019; Spitz et al., 2019; Wong et al., 2019; Brekke et al., 2020; Evin et al., 2021; Karanasios et al., 2021; Lima-Soares et al., 2022; Vehrs et al., 2023), only few studies have reported sex differences in AOP. Two previous studies (Jessee et al., 2016b; Mouser et al., 2017b) reported small sex differences in the brachial artery AOP that would be of little practical importance when using BFR during exercise. Similarly, Vehrs et al. (2023) recently reported non-significant sex differences in the AOP of the dominant and non-dominant legs. On the contrary, Tafuna’i et al. (2021) reported large and significant sex differences in the AOP of the legs. Although limb circumference explains some of the differences in AOP (Scott et al., 2015; McEwen et al., 2019; Hughes et al., 2017; Jessee et al., 2016a; Tafuna’i et al., 2021; Mattocks et al., 2018) between individuals, the large and significant sex differences in femoral artery AOP reported by Tafuna’i et al. (2021) occurred despite small and non-significant differences in thigh circumference.

Few studies have compared AOPs between the dominant and non-dominant limbs; in fact, few studies have identified the dominance of the limb of interest (Tafuna’i et al., 2021; Karanasios et al., 2021; Hughes et al., 2018a). In studies that have measured the AOP in both limbs, the differences in the AOP between the limbs have not been reported (Loenneke et al., 2012; Loenneke et al., 2013; Brandner et al., 2015; Loenneke et al., 2015; Barnett et al., 2016; Counts et al., 2016). Tafuna’i et al. (2021) recently reported a large and significant difference (21 ± 28 mmHg) in the AOP between the dominant and non-dominant legs of males despite similar circumferences of the two legs. Likewise, Crossley et al. (2019) reported a mean difference of over 40 mmHg in the AOP between the two legs. To the contrary, small non-significant differences (<6 mmHg) in AOP between the two legs have previously been reported (Evin et al., 2021; Vehrs et al., 2023). Based on the few studies that have reported AOP values of the paired limbs, further research comparing the AOP of the dominant and non-dominant limbs is warranted.

The differences between HHDOP and US measures of AOP, between males and females and between the dominant and non-dominant limbs, remain unclear. Thus, the primary purpose of this study was to compare US and HHDOP measures of AOP of the upper and lower limbs of young healthy males and females to evaluate the ability of HHDOP to measure AOP at both high (legs) and low (arms) cuff pressures.

## Materials and methods

### Participants

Previously unreported data from a total of 60 physically active and apparently healthy young adults, 19–28 years of age, who voluntarily participated in other studies were used in this study. To minimize the effects of hormone variability on some of the variables measured in the prior studies, females were asked to participate during the first 14 days of their menstrual cycle. We compared US and HHDOP measures of AOP of the brachial artery (15 males and 15 females) and femoral artery (15 males and 15 females) of the dominant and non-dominant limbs. Individuals who self-reported risk factors for cardiovascular disease or thromboembolism, with a diagnosis of or being treated for cardiovascular disease, renal disease, diabetes, or hypertension, or currently pregnant or less than 6 months postpartum (Barnett et al., 2016; Jessee et al., 2016b; Mattocks et al., 2017; Sieljacks et al., 2018) were excluded from participation in the study. Participants were instructed to refrain from eating during the 2 h prior to their participation, consuming caffeine during the previous 8 h, and vigorous physical activity during the 24 h prior to their participation (Mattocks et al., 2017; Sieljacks et al., 2018). After the methods, expectations, risks, and benefits of the study were explained to them, each participant voluntarily provided written informed consent.

### Procedures

All measurements for each participant were completed in a single visit to the lab. Participants’ body mass index (BMI; kg/m^2^) was calculated using height (cm) measured using a calibrated wall-mounted stadiometer scale (SECA Model 264; SECA, Chino, CA, United States) and body mass (kg) measured using a digital scale (Ohaus Model CD-33, Ohaus Corporation, Pine Brook, NJ, United States). Each participant self-reported arm and leg dominance by responding to the question, “If you were to throw (or kick) a ball, which arm (or leg) would you use to throw (or kick) the ball?” (van Melick et al., 2017). The volume (m^3^) of the upper arm and thigh was calculated using the formula describing the volume of a truncated cone (Jones and Pearson, 1969; Katch et al., 1973; Katch and Katch, 1974; Stranden, 1981; Perrin and Guez, 2000). To compute the volume of the upper arm, we used circumference measurements taken at the crease at the junction of the anterior border of the deltoid and the biceps brachii and the proximal border of the olecranon process of the ulna and the distance between the two circumferences. Measurements of the arm were taken with the arm extended to the side and parallel to the floor with the elbow at 0°. The participants were also asked to flex their elbow, and the point of the largest circumference was marked with a pen. A circumference measurement was taken at this location with the arm in a relaxed and extended position. We partitioned the thigh into two segments by measuring circumferences (cm) at three locations: the upper thigh at the level of the gluteal fold, lower thigh just above the proximal border of the patella, and mid-thigh one-third the distance between the upper and lower measurements. To compute the volume of the thigh, we also used the distances between the upper and mid-thigh circumferences and between the mid- and lower-thigh circumferences. All measurements of the thigh were taken in the standing position while bearing weight on the opposite leg. All circumferences of the arms and thighs were measured in triplicate using a spring-loaded Gullick measuring tape on both the dominant and non-dominant limbs. If two measurements were the same, that value was recorded; otherwise, an average of all three measurements was used.

Following anthropometric measurements, the participant sat on a patient table in a semi-reclined position with the legs extended (0° knee extension) and supported. After resting quietly for 5 min, resting HR and blood pressure were measured in duplicate using an automated blood pressure monitor (OMRON BP7200, OMRON Healthcare Inc.), and the average of the two values was recorded. Mean arterial pressure (MAP) was calculated from systolic (SBP) and diastolic (DBP) blood pressures.

### Measurement of arterial occlusion pressure

To measure AOP, a nylon pneumatic cuff (SC10: 11 × 85 cm; Hokanson, Belleview, WA; https://hokansonvascular.com) was placed around either the upper arm or the upper region of the thigh. An E-20 rapid cuff inflator (Hokanson, Bellevue, WA, United States) was used to inflate the cuff using a continuous cuff inflation protocol. The cuff was initially inflated to 50 mmHg and then gradually increased at a rate of 10 mmHg/10 s until arterial blood flow or pulse waves were no longer visible or pulse sounds were no longer audible as described below. The AOP of the brachial and femoral arteries was measured once on the dominant limb and once on the non-dominant limb in a random order separated by a 5 min rest period during which time the cuff was deflated and removed from the limb. Yet unpublished data from our lab indicated that mean differences, maximum differences, and correlations between two within-day trials for US (2.7 ± 6.4 mmHg, 14 mmHg, R = 0.826) and HHDOP (4.2 ± 4.7 mmHg, 13 mmHg, R = 0.897) were comparable. A previous study reported high test–retest (ICC = 0.858–0.900) and interrater reliability (ICC = 0.894–0.984) when using an HHDOP to measure AOP between three investigators and three body positions (Karanasios et al., 2021). As such, in this study, we did not assess reliability of multiple US or HHDOP measurements of AOP.

During each measurement, AOP was measured simultaneously using a US and an HHDOP. Doppler ultrasound measurements of AOP were performed using a Doppler ultrasound probe (9 MHz; 55 mm) and a LOGIQe ultrasound machine (GE Healthcare). When determining the AOP of the brachial artery, we monitored blood flow through the radial artery just below the elbow using color flow and Doppler (pulse wave) modes of the US and near the styloid process of the radius near the wrist using an HHDOP device (8 MHz probe, DigiDop II, Model 770R, Newman Medical, Arvada, CO, United States). When determining the AOP of the femoral artery, we monitored blood flow through the superficial femoral artery just distal to the cuff on the thigh with US and in the anterior tibial artery near the ankle using the HHDOP. The AOP of both arteries was defined as the lowest pressure at which the color flow and pulse waves were no longer visible using the US, and pulse sounds were no longer audible using the HHDOP.

### Data analysis

The statistical analyses of data were performed using Statistical Analysis System version 9.4 (SAS Inc., Cary, North Carolina, United States). The variables of interest in this study were age, height, body mass, BMI, blood pressure, arm circumference, arm volume, and AOP. Differences in age, height, body mass, BMI, and resting blood pressure (SBP, DBP, and MAP) and HR between males and females were determined using independent t-tests. Sex differences in the circumference and volume of the DOM and NDOM limbs (arms and legs) were also determined using independent *t*-tests. We also analyzed the data for differences in arm and leg circumference and volume between the dominant and non-dominant limbs within males and within females using a paired *t*-test. Because we performed multiple tests in each of these analyses, we chose a Bonferroni corrected critical *t*-value to maintain a family-wise alpha level of 0.05. We reported significant sex differences and significant differences between the dominant and non-dominant limbs if the test *t*-value was greater than the adjusted two-tailed critical *t*-value of 2.80 (*p* = 0.00625).

The primary variable of interest in this study was AOP measured using the US and HHDOP in the dominant and non-dominant arms and legs. We used a mixed model ANOVA to analyze the AOP measurements using the US and HHDOP in the dominant and non-dominant limbs of males and females. Sex (male/female) was considered a between-group factor, and dominance (dominant/non-dominant) and method (US/HHDOP) were considered within-group factors. Separate analyses were performed for the arms and legs. The mixed model ANOVA appropriately accounts for multiples sources of variability. We evaluated the main effects of limb dominance (dominant and non-dominant), method (US and HHDOP), and sex (male and female) as well as all two-way and three-way interactions. Individual comparisons of US and HHDOP measures of AOP within or between dominant and non-dominant limbs, and within or between males and females were performed while using a Bonferroni correction for multiple tests to maintain a family-wise *p*-value of 0.05. The influence of limb circumference, limb volume, gender, and blood pressure on the AOP was evaluated using regression analysis.

The agreement between US and HHDOP measures of AOP was evaluated using Bland–Altman analysis (Bland and Altman, 1986). Separate analyses were performed for the arms and legs. We calculated the mean difference (bias) between US and HHDOP measures of AOP by subtracting the US measure of AOP from the HHDOP measure of AOP so a negative bias represented an underestimation of AOP by the HHDOP. The limits of agreement (LOAs) for the HHDOP measures of AOP were calculated as the average bias ±1.96 SD (Bland and Altman, 1986). Bland–Altman plots were created by plotting the bias between the US and HHDOP measures of AOP (*y*-axis) and the mean of US and HHDOP measures of AOP (*x*-axis). The ideal agreement between US and HHDOP measures of AOP would result in a Bland–Altman plot with a mean bias (*y*-axis) that is not significantly different from zero and a slope of the line-of-best fit through the data being equivalent to zero, indicating that the bias is consistent across the range of AOP values (*x*-axis).

## Results

Male participants were significantly (*p* < 0.05) older (23.3 ± 1.7 yr; 21.6 ± 1.2 yr), taller (179.0 ± 6.5 cm; 168.9 ± 6.3 cm), and heavier (81.3 ± 13.7 kg; 66.9 ± 11.7 kg) and had higher resting SBPs (122.8 ± 9.8 mmHg; 112.5 ± 7.4 mmHg) and MAPs (93.8 ± 7.3 mmHg; 88.1 ± 6.0 mmHg) compared to the female participants, respectively. There were no significant sex differences in BMI (25.4 ± 4.4 kg/m^2^; 23.4 ± 3.9 kg/m^2^), resting DBP (79.4 ± 7.5 mmHg; 75.8 ± 6.5 mmHg), or resting HR (73.0 ± 11.2 bpm; 73.6 ± 10.9 bpm). There were no significant differences in the circumferences or volumes of the DOM and NDOM arms or legs between males and females or between the DOM and NDOM arms (Table 1) or legs (Table 2) within males and within females.

**TABLE 1:** Arm dimensions.

	Male	Female	Difference	*p*-value
Arm circumference (cm)				
Dominant arm	29.5 ± 4.1	26.7 ± 2.9	2.7 ± 1.3	0.041
Non-dominant arm	29.5 ± 4.1	26.4 ± 2.7	3.1 ± 1.3	0.020
Difference	0.05 ± 1.5	0.3 ± 1.02		
	*p* = 0.975	*p* = 0.787		
Arm volume (m^3^)				
Dominant arm	0.041 ± 0.014	0.031 ± 0.008	0.010 ± 0.004	0.027
Non-Dominant arm	0.041 ± 0.013	0.033 ± 0.010	0.008 ± 0.004	0.079
Difference	0.000 ± 0.005	0.002 ± 0.004		
	*p* = 0.887	*p* = 0.677		

Values are mean ± SD. No significant differences (*p*-values > Bonferroni adjusted *p* = 0.006) were found in arm circumference or volume between the dominant and non-dominant arms in males and females or between males and females.

**TABLE 2:** Leg dimensions.

	Male	Female	Difference	*p*-value
Thigh circumference (cm)				
Dominant leg	59.9 ± 6.1	56.2 ± 3.1	3.7 ± 1.8	0.043
Non-dominant leg	59.6 ± 5.7	55.7 ± 3.4	3.8 ± 1.7	0.033
Difference	0.4 ± 2.2	0.5 ± 1.2		
	*p* = 0.403	*p* = 0.167		
Thigh volume (m^3^)				
Dominant leg	0.241 ± 0.058	0.207 ± 0.033	0.033 ± 0.017	0.064
Non-Dominant leg	0.230 ± 0.053	0.205 ± 0.029	0.025 ± 0.016	0.126
Difference	0.010 ± 0.021	0.002 ± 0.011		
	*p* = 0.121	*p* = 0.778		

Values are mean ± SD. No significant differences (*p*-values > Bonferroni adjusted *p* = 0.006) were found in leg circumference or volume in the dominant and non-dominant legs between males and females or between the dominant and non-dominant legs in males or females.

Table 3 includes US and HHDOP measures of AOP in the arms. The initial ANOVA indicated a significant overall main effect for sex (*p* = 0.002) with males (123.1 ± 9.8 mmHg) having a higher overall AOP compared to females (111.9 ± 9.2 mmHg). There were no significant overall main effects for method (US vs. HHDOP; *p* = 0.069) or leg dominance (dominant vs. non-dominant; *p* = 0.231) and no significant two-way interactions. Because the three-way interaction (method × dominance × gender) approached significance (*p* = 0.054), we performed multiple comparisons using a Bonferroni adjusted *p*-value to evaluate the effects of limb dominance, method, and sex on each other while maintaining a family-wise *p*-value of 0.05. The small differences between US and HHDOP measures of AOP were not significant (*p* > 0.05) within the dominant or non-dominant arms of males or females. Likewise, the US and HHDOP measures of AOP were not significantly different (*p* > 0.05) between the dominant or non-dominant arms of males or females. The sex differences in the US and HHDOP measures of AOP were not significant in the dominant arm (10.1 ± 3.6 mmHg; 10.9 ± 3.7 mmHg) but were in the non-dominant arm (13.0 ± 3.4 mmHg; 10.7 ± 3.4 mmHg), respectively.

**TABLE 3:** Ultrasound and Doppler measurements of brachial artery occlusion pressure.

	Ultrasound	Doppler	Difference	*p*-value
Males				
Dominant arm	123.7 ± 9.4	123.3 ± 9.2	0.5 ± 1.9	0.371
Non-dominant arm	123.5 ± 10.4	121.9 ± 10.8	1.6 ± 2.1	0.010
Difference	0.2 ± 3.6	1.3 ± 3.7		
	*p* = 0.901	*p* = 0.378		
Females				
Dominant arm	113.6 ± 10.4	112.4 ± 11.2	1.2 ± 2.4	0.076
Non-dominant arm	110.5 ± 8.1	111.2 ± 7.4	0.7 ± 4.1	0.537
Difference	3.1 ± 7.5	1.2 ± 7.7		
	*p* = 0.137	*p* = 0.553		
Sex differences				
Dominant arm	10.1 ± 3.6	10.9 ± 3.7		
	*p* = 0.009	*p* = 0.007		
Non-dominant arm	13.0 ± 3.4*	10.7 ± 3.4*		
	*p* = 0.001	*p* = 0.004		

* = significant sex differences (*p*-values < Bonferroni adjusted *p* = 0.004) in the non-dominant arm when AOP is measured using ultrasound and the handheld Doppler. No significant differences were found within or between the dominant and non-dominant arms when AOP is measured using an ultrasound or the handheld Doppler in males or females.

Table 4 includes US and HHDOP measures of AOP in the legs. The initial analysis indicated a significant overall main effect (*p* < 0.0001) for the method (US vs. HHDOP) with the HHDOP (203.3 ± 45.4 mmHg) yielding lower overall AOP values compared to US (206.2 ± 45.4 mmHg) measures of AOP. There were no significant main effects for sex (*p* = 0.973) or leg dominance (dominant vs. non-dominant; *p* = 0.675) and no significant two or three-way interactions. Additional comparisons revealed that the small differences between US and HHDOP measures of AOP in the dominant leg of males and females were significant (*p* = 0.0001). The smaller differences between the US and HHDOP measures of AOP in the non-dominant legs were not significant. There were no significant (*p* > 0.05) differences in AOP between the dominant and non-dominant legs of males or females when using the US or HHDOP. The sex differences in US and HHDOP measures of AOP in the dominant leg (6.6 ± 17.8 mmHg; 6.8 ± 17.7 mmHg) and non-dominant leg (8.3 ± 15.9 mmHg; 7.2 ± 16.1 mmHg), respectively, were not significant.

**TABLE 4:** Ultrasound and Doppler measurements of femoral artery occlusion pressure.

	Ultrasound	Doppler	Difference	*p*-value
Males				
Dominant leg	204.7 ± 23.7	201.1 ± 22.9	3.6 ± 2.7	0.0001*
Non-dominant leg	208.5 ± 33.3	205.6 ± 33.8	2.9 ± 3.5	0.007
Difference	3.8 ± 10.5	4.4 ± 10.6		
	*p* = 0.670	*p* = 0.630		
Females				
Dominant leg	211.3 ± 64.9	208.0 ± 64.6	3.3 ± 1.9	0.0001*
Non-dominant leg	200.2 ± 52.0	198.4 ± 52.6	1.8 ± 2.9	0.032
Difference	11.1 ± 21.4	9.6 ± 21.5		
	*p* = 0.350	*p* = 0.420		
Sex differences				
Dominant leg	6.6 ± 17.8	6.8 ± 17.7		
	*p* = 0.714	*p* = 0.701		
Non-dominant leg	8.3 ± 15.9	7.2 ± 16.1		
	*p* = 0.606	*p* = 0.659		

* = significance differences (*p*-values < Bonferroni adjusted *p* = 0.004) between ultrasound and handheld Doppler measures of AOP in the dominant legs of males and females. Differences in AOP between the dominant and non-dominant legs within males and females were not significantly different when measured using an ultrasound or the handheld Doppler device. No significant sex differences in the AOP in the dominant leg or non-dominant leg were observed.

Regression analysis revealed that after arm circumference (*r* = 0.731, *p* = 0.001) and MAP (*r* = 0.859; *p* = 0.001) entered the equation, SBP, DBP, arm volume, and sex were not significant predictors of brachial artery AOP. In another regression analysis, no independent variables entered the equation as significant predictors of femoral artery AOP.

**FIGURE 1:**
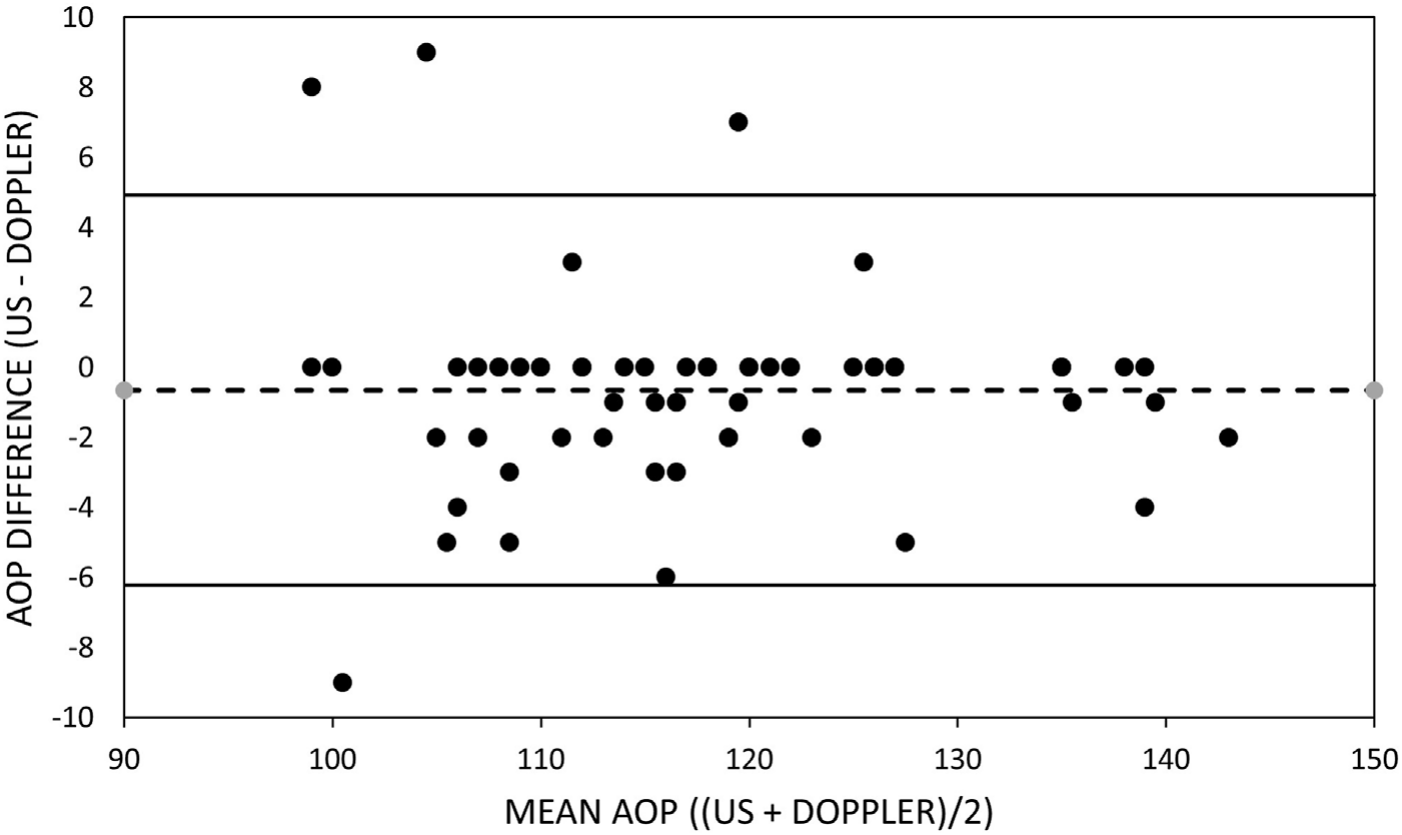
Bland–Altman plot of brachial artery occlusion pressure measurements. Bland–Altman plots of the ultrasound and handheld Doppler measures of brachial artery arterial occlusion pressure. Upper and lower dashed lines represent the limits of agreement. Middle dashed line represents the bias.

**FIGURE 2:**
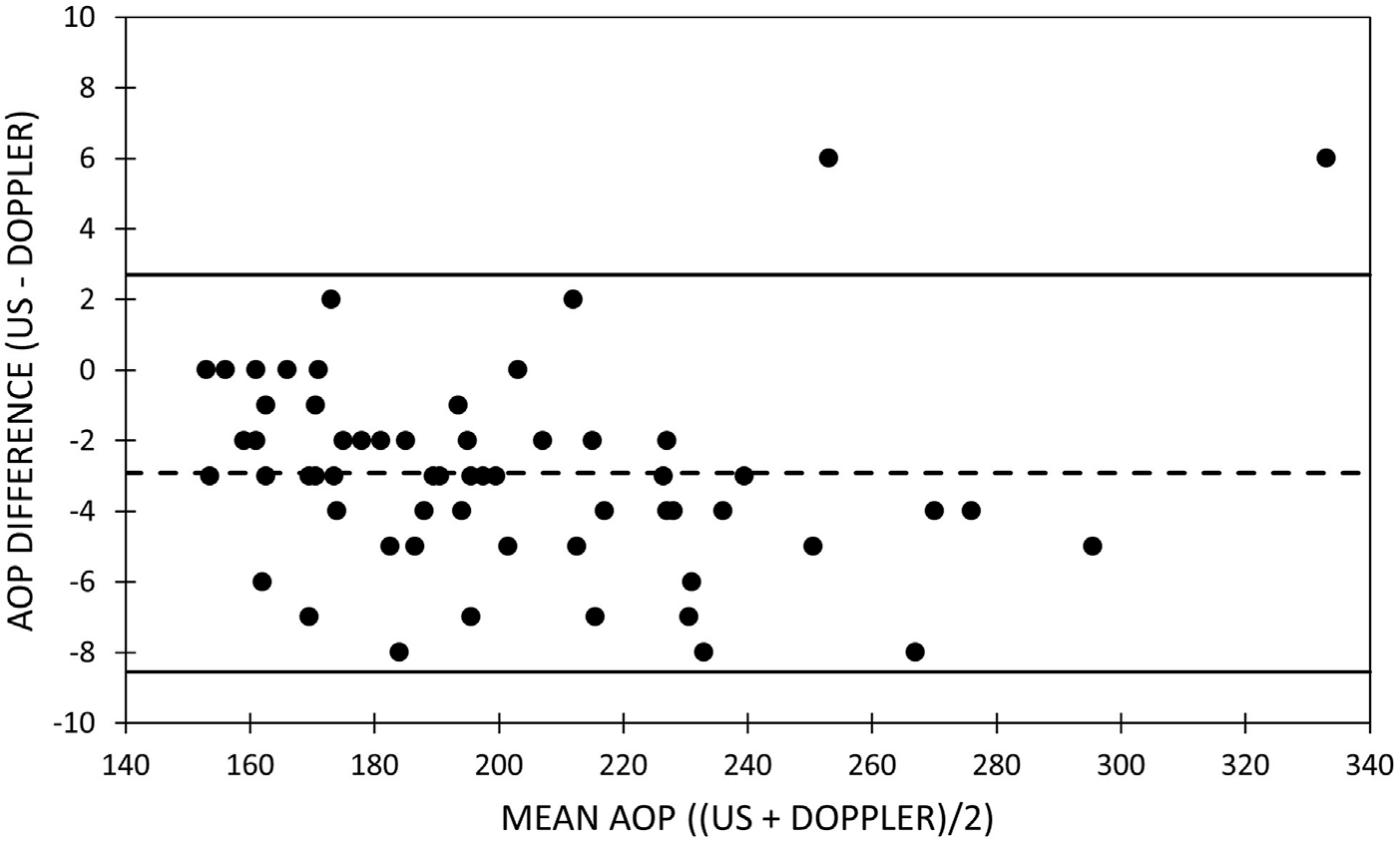
Bland–Altman plot of femoral artery occlusion pressure measurements. Bland–Altman plots of the ultrasound and handheld Doppler measures of femoral artery arterial occlusion pressure. Upper and lower dashed lines represent the limits of agreement. Middle dashed line represents the bias.

Table 5 includes the mean bias, LOA, and slope of the line of best fit through the data in the Bland–Altman plots for the arms and legs (Figures 1, 2). The mean bias of −0.65 mmHg between US and HHDOP measures of AOP in the arms was not significantly different from zero (*p* = 0.81), and the slope (−0.0093) of the line of best fit through the data was not significantly different from zero (Figure 1). The mean bias of −2.93 mmHg between US and HHDOP measures of AOP in the legs was significantly different from zero (*p* = 0.001), and the slope (0.0018) of the line of best fit through the data was not significantly different from zero (Figure 2).

**TABLE 5:** Bias and limits of agreement used to generate Bland–Altman plots.

	Bias	Limits of agreement	Slope
Arms	−0.65	±5.56 (−6.21 to 4.91)	−0.0093
Legs	−2.93	±5.58 (−8.51 to 2.65)	0.0018

Bias = average difference between HHDOP and US measures of AOP. Limits of agreement = ±1.96 SD of bias (lower and upper limits of agreement). Slope = slope of the line of best fit through data.

## Discussion

The results of this research contribute to our current understanding of measuring AOP using a relatively inexpensive handheld Doppler. We report small differences in the simultaneous measures of AOP using the US and HHDOP in the dominant and non-dominant arms and legs of young adult males and females that are of little practical importance. We also report no significant differences between the dominant and non-dominant arms and legs in both males and females when using either the US or HHDOP to measure AOP. Sex differences in AOP existed in both the arms and legs but were only large enough to be significant in the arms.

### Measuring arterial occlusion pressure with a handheld Doppler

Arterial occlusion pressure was measured in the dominant and non-dominant arms and legs simultaneously with a “gold standard” US and a HHDOP ‘field’ device. When measuring the AOP of the brachial artery, the small overall mean difference (−0.65 ± 2.8 mmHg) between the US and HHDOP measures of AOP was not significant. This pattern was consistent within and between the dominant and non-dominant arms of males and females (Table 3). The bias between US and HHDOP measures of AOP were within ±5 mmHg in 92% of the measurements, and the largest bias was only 9 mmHg (Table 5; Figure 1).

We report a small, but statistically significant overall mean difference (−2.92 ± 2.8 mmHg) observed between US and HHDOP measures of femoral artery AOP (Table 4). The differences between US and HHDOP measures of AOP appear to be slightly larger in males than in females and slightly larger in the dominant leg than in the non-dominant leg. The bias between US and HHDOP measures of AOP in the leg was within ±5 mmHg in 82% of the measurements, and the largest bias was only 8 mmHg (Table 5; Figure 2).

Other studies (Karanasios et al., 2021; Lima-Soares et al., 2022; Barrett et al., 2023) have also used a HHDOP to measure AOP, but these studies did not compare HHDOP measures of AOP to those of a “gold standard.” Our data comparing US and HHDOP measures of AOP concur with those of Laurentino et al. (2020) who recently reported similar US and HHDOP measures of AOP in the legs of young adult males. The mean AOP values reported by Laurentino et al. using the US (135 ± 17 mmHg) and HHDOP (133 ± 18 mmHg) were much smaller than the AOP values reported in this study. This can be attributed to the differences in cuff size used in this study (11 cm) vs. the 17.5 cm cuffs used in Laurentino et al.’s study. Although Laurentino et al. (2020) reported similar US and HHDOP mean AOP values, they reported Bland–Altman plots with biases that approached 20 mmHg. The differences in the biases may be due to the methodology. In this study, we measured AOP simultaneously with the US and HHDOP, whereas Laurentino et al. (2020) made separate measurements at different times. In this study, we measured AOP simultaneously in order to reduce the likelihood of differences in AOP measured using the two devices being due to measurements taken at different times. In this study, the small differences in US and HHDOP measurements of AOP could be due to the differences in the location of the measurements. AOP using the US was measured at the superficial femoral artery close to the occlusion cuff, whereas the HHDOP was used to measure AOP further downstream at the anterior tibial artery at the ankle. Blood flow and the velocity of blood flow through the large superficial femoral artery are greater than in the smaller anterior tibial artery, which may contribute to the ability to detect blood flow. Nevertheless, Laurentino et al. (2020) measured AOP using the US and HHDOP at the same locations and reported no significant differences in the two measurements of AOP. Although there are differences in the size of the vessel and velocity and volume of blood flow at the two locations, when measurements are taken simultaneously, the small differences in the AOP measurement reported in this study are likely due to differences in technology. When using the US, AOP is determined using two criteria (the absence of color flow and the absence of a pulse wave), whereas when using the HHDOP, AOP is determined merely by the absence of an audible pulse wave.

Differences between US and HHDOP measures of AOP of the brachial and femoral arteries of the magnitude reported in this study would not increase the risk or affect the effectiveness of using BFR during exercise. For example, using the overall average AOP measured in the brachial (≈118 mmHg) and femoral (≈206 mmHg) arteries and the recommended cuff pressure when using BFR during exercise of 40%–80% of the limbs AOP (Scott et al., 2015; Hughes et al., 2017; McEwen et al., 2019; Patterson et al., 2019; Cognetti et al., 2022), a difference in the AOP of 8–9 mmHg when using an HHDOP would result in a cuff inflation pressure during BFR that is within 3–7 mmHg of that determined when using a US to measure AOP. Therefore, we suggested that the differences in US and HHDOP measures of AOP reported in this study are of little practical significance when using BFR in the field.

### Sex and limb differences in arterial occlusion pressure

In this study, sex differences in brachial artery AOP existed in both the dominant and non-dominant arms but only rose to a level of significance in the non-dominant arm (Table 3). Because limb circumference is a key determinant of AOP (Scott et al., 2015; McEwen et al., 2019; Nascimento et al., 2019; Tafuna’i et al., 2021; Mattocks et al., 2018; Hughes et al., 2018b), the differences in AOP between males and females could be attributed to differences in the limb’s circumference. Nevertheless, in this study, the arm circumferences and volumes (Table 1) were similar in males and females. In this study, limb circumference (*r* = 0.731) and MAP (*r* = 0.859) were the variables most highly correlated with brachial artery AOP and the only two variables that entered into a regression analysis to predict AOP. Interestingly, compared to their female counterparts, males in this study had higher MAP values. Previous studies (Jessee et al., 2016b; Mouser et al., 2017b) have also reported significant sex differences in brachial artery AOP. The sex differences reported by Jessee et al. (2016b), although statistically significant, were not large and of little importance. The larger sex differences (approximately 12–15 mmHg) reported by Mouser et al. (2017b) were also statistically significant. The sex differences in AOP were unexplained in both studies (Jessee et al., 2016b; Mouser et al., 2017b).

In this study, the sex differences in the femoral artery AOP in the dominant and non-dominant legs as measured by using the US and HHDOP were not statistically significant (Table 4). In this study, thigh circumference and volume were similar between the dominant and non-dominant legs and between males and females (Table 2). This is consistent with a recent study reporting no significant differences in femoral artery AOP between males and females in either the dominant or non-dominant legs (Vehrs et al., 2023). On the contrary, Tafuna’i et al. (2021) recently reported large significant differences in femoral artery AOP between males and females despite small and nonsignificant mean differences in limb circumference and volume. In this study, leg circumference, leg volume, blood pressures, and sex did not enter the regression analysis to predict femoral artery AOP. Although we would have expected leg circumference to be a significant independent variable predictive of AOP, there was a low, non-significant correlation (r = 0.204; *p* = 0.059) between leg circumference and AOP. One possible explanation is that similar to data reported in a previous study (see Figure 1 of the work of Tafuna’i et al. (2021)), there is a wide range of AOP values for a given leg circumference. Thus, factors other than leg circumference influence AOP.

In this study, the mean differences in AOP between the dominant and non-dominant limbs were small and of little practical significance (Tables 3, 4). Our data concur with previously reported data (Evin et al., 2021; Vehrs et al., 2023) indicating non-significant differences in AOP between the two legs. To assume that small differences in AOP between the dominant and non-dominant limbs persisted across individuals would be a gross misinterpretation. For example, in this study, although the mean differences in AOP between the dominant and non-dominant legs were relatively small, the differences between the dominant and non-dominant legs for an individual ranged from 2 mmHg up to 107 mmHg. This range of differences between the dominant and non-dominant legs is similar to that previously reported by Tafuna’i et al. (2021). A small recent pilot study (Citherlet et al., 2022) that identified the legs as right and left (rather than dominant and non-dominant) reported differences in leg AOP ranging from 8 mmHg up to 76 mmHg. The difference between the dominant and non-dominant arms in this study was much smaller than that in the legs, ranging from 0 mmHg up to 18 mmHg. The smaller range of differences in the upper arm is likely due to the smaller circumference of the arm, lower AOP, and closer proximity to the heart and similarity to resting brachial systolic blood pressure. Factors such as systolic blood pressure or mean arterial pressure (Loenneke et al., 2015; Brown et al., 2018; Vehrs et al., 2023) or other unknown factors could potentially explain differences in AOP between individuals, between males and females, and between dominant and non-dominant limbs. Evaluating factors such as the diameter of the vessel, resting arterial blood flow, vascular health, fitness level, and training status may lead to a better understanding of individual differences in AOP.

### Study strengths and limitations

The strengths of this study are that comparisons were made between simultaneous measures of AOP using a US and an HHDOP in and between the dominant and non-dominant arms and legs of males and females. This study is not without limitations. This study included young, apparently healthy, physically active coeds. The results of this study may not apply to other segments of the population (e.g., age, health status, training status). In this study, we used a research/clinical-grade cuff inflation system and a nylon 11 cm cuff for all measurements of AOP. Those who use BFR during exercise likely use other types and styles of cuffs and other methods to inflate the cuffs. The results of this study may not apply to the use of different cuffs and cuff inflation systems.

### Directions for future studies

Differences in AOP measurements can occur when using cuffs of different sizes, bladder lengths, bladder designs, and cuff material, methods to inflate the cuff, and devices to measure AOP. Evaluations of other handheld devices (e.g., pulse oximeters) to measure AOP; other methods to inflate the cuff (e.g., manually inflating the cuff with a sphygmomanometer); and other styles of cuff (e.g., single- vs. multichambered cuff) are warranted. We encourage future studies to report sex differences in AOP and comparisons between the dominant and non-dominant limbs, continue to explore factors that could contribute to differences in AOP, and evaluate the effect of training status and training interventions on AOP.

## Conclusion

Given that any differences between US and HHDOP measures of AOP in the arms and legs were small and of little practical significance, a HHDOP is a viable alternative to the use of expensive US devices for the measurement of AOP. As such, a HHDOP can be used to measure AOP by practitioners for the safe and effective use of BFR. Due to the potentially large differences in AOP between dominant and non-dominant limbs, particularly in the legs, AOP should be measured in each limb prior to using BFR during exercise. Because AOP varies with the characteristics of the blood flow restriction cuffs (width, bladder design, and material), the cuff used to apply BFR during exercise should be the same cuff used to measure AOP.

## Data Availability

The raw data supporting the conclusion of this article will be made available by the authors, without undue reservation.
