# Supplementary material for: Use of a handheld Doppler to measure brachial and femoral artery occlusion pressure
Source: Front Physiol. 2023 Aug 17;14:1239582. doi: 10.3389/fphys.2023.1239582 (PMC10470651; doi:10.3389/fphys.2023.1239582)
Supplement: Supplementary file 1 [file Table1.DOCX]

Table 1. Arm Dimensions

Males Females Difference *p*-value

Arm Circumference (cm)

Dominant Arm 29.5 ± 4.1 26.7 ± 2.9 2.7 ± 1.3 0.041

Non-dominant Arm 29.5 ± 4.1 26.4 ± 2.7 3.1 ± 1.3 0.020

Difference 0.05 ± 1.5 0.3 ± 1.02

*p* = 0.975 *p* = 0.787

Arm Volume (m^3^)

Dominant Arm 0.041 ± 0.014 0.031 ± 0.008 0.010 ± 0.004 0.027

Non-Dominant Arm 0.041 ± 0.013 0.033 ± 0.010 0.008 ± 0.004 0.079

Difference 0.000 ± 0.005 0.002 ± 0.004

*p* = 0.887 *p* = 0.677

Values are mean ± SD. No significant differences (*p*-values > Bonferroni adjusted *p* = 0.006) in arm circumference or volume between the dominant and non-dominant arms in males and females or between males and females.
