# Supplementary material for: Use of a handheld Doppler to measure brachial and femoral artery occlusion pressure
Source: Front Physiol. 2023 Aug 17;14:1239582. doi: 10.3389/fphys.2023.1239582 (PMC10470651; doi:10.3389/fphys.2023.1239582)
Supplement: Supplementary file 2 [file Table2.DOCX]

Table 2. Leg Dimensions

Males Females Difference *p*-value

Thigh Circumference (cm)

Dominant Leg 59.9 ± 6.1 56.2 ± 3.1 3.7 ± 1.8 0.043

Non-dominant Leg 59.6 ± 5.7 55.7 ± 3.4 3.8 ± 1.7 0.033

Difference 0.4 ± 2.2 0.5 ± 1.2

*p* = 0.403 *p* = 0.167

Thigh Volume (m^3^)

Dominant Leg 0.241 ± 0.058 0.207 ± 0.033 0.033 ± 0.017 0.064

Non-Dominant Leg 0.230 ± 0.053 0.205 ± 0.029 0.025 ± 0.016 0.126

Difference 0.010 ± 0.021 0.002 ± 0.011

*p* = 0.121 *p* = 0.778

Values are mean ± SD. No significant differences (*p*-values > Bonferroni adjusted *p* = 0.006) in leg circumference or volume in the dominant and non-dominant legs between males and females or between the dominant and non-dominant legs in males or females.
