# Supplementary material for: Use of a handheld Doppler to measure brachial and femoral artery occlusion pressure
Source: Front Physiol. 2023 Aug 17;14:1239582. doi: 10.3389/fphys.2023.1239582 (PMC10470651; doi:10.3389/fphys.2023.1239582)
Supplement: Supplementary file 3 [file Table5.DOCX]

Table 5. Bias and Limits of Agreement Used to Generate Bland-Altman Plots

___________________________________________________________

Bias Limits of Agreement Slope

___________________________________________________________

Arms -0.65 ±5.56 (-6.21 to 4.91) -0.0093

Legs -2.93 ±5.58 (-8.51 to 2.65) 0.0018

__________________________________________________________

Bias = average difference between HHDOP and US measures of AOP.

Limits of Agreement = ±1.96 SD of bias (lower and upper limits of agreement).

Slope = slope of the line of best fit through data.
