# Supplementary material for: Use of a handheld Doppler to measure brachial and femoral artery occlusion pressure
Source: Front Physiol. 2023 Aug 17;14:1239582. doi: 10.3389/fphys.2023.1239582 (PMC10470651; doi:10.3389/fphys.2023.1239582)
Supplement: Supplementary file 4 [file Table3.DOCX]

Table 3. Ultrasound and Doppler Measurements of Brachial Artery Occlusion Pressure.

Ultrasound Doppler Difference *p*-value

MALES

Dominant Arm 123.7 ± 9.4 123.3 ± 9.2 0.5 ± 1.9 0.371

Non-dominant Arm 123.5 ± 10.4 121.9 ± 10.8 1.6 ± 2.1 0.010

Difference 0.2 ± 3.6 1.3 ± 3.7

*p* = 0.901 *p* = 0.378

FEMALES

Dominant Arm 113.6 ± 10.4 112.4 ± 11.2 1.2 ± 2.4 0.076

Non-dominant Arm 110.5 ± 8.1 111.2 ± 7.4 0.7 ± 4.1 0.537

Difference 3.1 ± 7.5 1.2 ± 7.7

*p* = 0.137 *p* = 0.553

SEX DIFFERENCES

Dominant Arm 10.1 ± 3.6 10.9 ± 3.7

*p* = 0.009 *p* = 0.007

Non-dominant Arm 13.0 ± 3.4 * 10.7 ± 3.4 *

*p* = 0.001 *p* = 0.004

* = significant sex differences (p-values < Bonferroni adjusted *p* = 0.004) in the non-dominant arm when AOP is measured using ultrasound and the handheld Doppler. No significant differences within or between the dominant and non-dominant arms when AOP is measured using ultrasound or the handheld Doppler in males or females.
