# Supplementary material for: Use of a handheld Doppler to measure brachial and femoral artery occlusion pressure
Source: Front Physiol. 2023 Aug 17;14:1239582. doi: 10.3389/fphys.2023.1239582 (PMC10470651; doi:10.3389/fphys.2023.1239582)
Supplement: Supplementary file 5 [file Table4.DOCX]

Table 4. Ultrasound and Doppler Measurements of Femoral Artery Occlusion Pressure.

______________________________________________________________________________

Ultrasound Doppler Difference *p*-value

MALES

Dominant Leg 204.7 ± 23.7 201.1 ± 22.9 3.6 ± 2.7 0.0001 *

Non-dominant Leg 208.5 ± 33.3 205.6 ± 33.8 2.9 ± 3.5 0.007

Difference 3.8 ± 10.5 4.4 ± 10.6

*p* = 0.670 *p* = 0.630

FEMALES

Dominant Leg 211.3 ± 64.9 208.0 ± 64.6 3.3 ± 1.9 0.0001 *

Non-dominant Leg 200.2 ± 52.0 198.4 ± 52.6 1.8 ± 2.9 0.032

Difference 11.1 ± 21.4 9.6 ± 21.5

*p* = 0.350 *p* = 0.420

SEX DIFFERENCES

Dominant Leg 6.6 ± 17.8 6.8 ± 17.7

*p* = 0.714 *p* = 0.701

Non-dominant Leg 8.3 ± 15.9 7.2 ± 16.1

*p* = 0.606 *p* = 0.659

______________________________________________________________________________

* = significance differences (p-values < Bonferroni adjusted *p* = 0.004) between ultrasound and handheld Doppler measures of AOP in the dominant legs of males and females. Differences in the AOP between the dominant and non-dominant legs within males and females were not significantly different when measured using ultrasound or the handheld doppler. No significant sex differences in the AOP in the dominant leg or non-dominant leg.
